# Adoption and Use of Digital Technologies among General Dental Practitioners in the Netherlands

**DOI:** 10.1371/journal.pone.0120725

**Published:** 2015-03-26

**Authors:** Marieke M. van der Zande, Ronald C. Gorter, Irene H. A. Aartman, Daniel Wismeijer

**Affiliations:** 1 Department of Social Dentistry and Behavioural Sciences, Academic Centre for Dentistry Amsterdam (ACTA), University of Amsterdam and VU University, Amsterdam, the Netherlands; 2 Department of Oral Implantology and Prosthetic Dentistry, Academic Centre for Dentistry Amsterdam (ACTA), University of Amsterdam and VU University, Amsterdam, the Netherlands; UNC School of Dentistry, University of North Carolina-Chapel Hill, UNITED STATES

## Abstract

**Objectives:**

To investigate (1) the degree of digital technology adoption among general dental practitioners, and to assess (2) which personal and practice factors are associated with technology use.

**Methods:**

A questionnaire was distributed among a stratified sample of 1000 general dental practitioners in the Netherlands, to measure the use of fifteen administrative, communicative, clinical and diagnostic technologies, as well as personal factors and dental practice characteristics.

**Results:**

The response rate was 31.3%; 65.1% replied to the questionnaire on paper and 34.9% online. Each specific digital technology was used by between 93.2% and 6.8% of the dentists. Administrative technologies were generally used by more dentists than clinical technologies. Dentists had adopted an average number of 6.3±2.3 technologies. 22.5% were low technology users (0 to 4 technologies), 46.2% were intermediate technology users (5 to 7 technologies) and 31.3% were high technology users (8 to12 technologies). High technology users more frequently had a specialization (p<0.001), were younger on average (p=0.024), and worked more hours per week (p=0.003) than low technology users, and invested more hours per year in professional activities (p=0.026) than intermediate technology users. High technology use was also more common for dentists working in practices with a higher average number of patients per year (p<0.001), with more dentists working in the practice (p<0.001) and with more staff (p<0.001).

**Conclusion:**

With few exceptions, all dentists use some or a substantial number of digital technologies. Technology use is associated with various patterns of person-specific factors, and is higher when working in larger dental practices. The findings provide insight into the current state of digital technology adoption in dental practices. Further exploration why some dentists are more reluctant to adopt technologies than others is valuable for the dental profession’s agility in adjusting to technological developments.

## Introduction

In dentistry, as in other professions, digital alternatives for existing work practices are continuously emerging. New digital technologies are already omnipresent in many aspects of the dental workflow [1–4]. Almost without fail, they are brought to dentists’ attention in conferences, correspondence, email, courses and advertisements. As with digital objects in other areas of life—for instance smartphones, music carriers, social media platforms, and car technology—their presence is felt in many areas of activity, and newer technologies are constantly competing with existing ones, the focus often lying on one specific technology or new model at a time. However, technologies are not used in isolation—they are used together with others, with each new technology being weighed against alternatives.

Everywhere, the use and adoption of digital technologies differs considerably from person to person and from organization to organization [5–11]. The characteristics of adopters and of technology itself have often been studied as factors that account for differences in adoption [5,8,9,11]. Adoption studies focus on technologies and innovations that are relatively new to their potential users, and are often based on the observation that, despite their great potential benefits, many innovations are not adopted to the extent expected. While individual and organizational differences are often explained by contextual factors such as organizational size and interactions between professional groups, these factors have received less attention in studies on technology use [12–15]. Usually, technological innovations are studied in contexts involving heterogeneous groups of individuals and relatively large organizations, which differ from most dental practices.

Several studies in dentistry have examined the degree of computerization in dental practices [2,16–18], computerization in relation to information seeking [19,20], and the adoption and diffusion of specific technologies among dental professionals [21–25]. While they provide important evidence on dental technology use, they do not create an overall picture of the present adoption and use of digital dental technologies or the factors underlying these. The aim of this study is therefore to investigate (1) the extent to which digital technologies are used, and in which combinations, and to assess (2) the person and practice characteristics associated with digital technology adoption.

## Materials and Methods

### Study sample

Data were collected between April and July 2013 using a questionnaire distributed among general dental practitioners in the Netherlands. The sample was selected from a panel of Dutch dentists who are regularly surveyed by the Royal Dutch Dental Association (KNMT). From the total population of 8698 dentists in the Netherlands with a registered practice or home address in 2012, a stratified random sample of 1000 general dental practitioners was drawn. The sample was stratified by age and gender of the respondents to ensure that it covered a representative sample of Dutch dentists. It included general dental practitioners as well as specialized dentists working in private or group practices or larger clinics and hospitals.

### Data collection

The questionnaire was developed based on interviews with experts in dentistry, dental education and dental technology which explored influential digital technologies in the dental field and factors that may influence their use. More details about the interview study are reported in an earlier paper [26]. The factors most often mentioned by the experts were compared with literature on technology adoption and use, and included if relevant on the basis of this comparison. The construction of the questionnaire was elaborately discussed between the first author, a sociologist, the second author, a psychologist working in dental education and the last author, a dentist and professor in implant dentistry, to ensure that different viewpoints were brought together. The initial construction of the questionnaire was further discussed with the third author, a methodologist, and with the coordinator of the panel studies. After repeated discussion and revision, a pilot was conducted among five dental practitioners. Based on the clarifications asked by pilot participants, the questionnaire was revised. One question, asking dentists about their own digital technology use compared to other dentists, was moved to the final part of the questionnaire. In addition to clarifying wording on some questions, answer categories were revised for three questions. The number of working hours per week was changed into working hours at chairside and non-chairside hours. The number of patients visiting the practice was given an open answer format instead of closed categories, and the number of hours used for professional activities was changed from hours per month to hours per year for two of the items. Finally, the revised questionnaire was reviewed by and discussed with the Royal Dutch Dental Association’s research committee (KNMT/COB), which evaluates research proposals and protocols for the Royal Dutch Dental Association (KNMT) panel studies.

Each respondent received a postal questionnaire, accompanied by a self-addressed pre-paid return envelope and an invitation letter. The invitation letter detailed the purpose of the study and provided each respondent with a unique login code to fill in the online questionnaire. Respondents either returned the paper version of the questionnaire or completed the questionnaire online depending on their choice. Non-respondents were sent a reminder by post, a second reminder by email three weeks later, and three weeks after the second reminder a sample of non-respondents was approached with a reminder by telephone. Both the distribution of the questionnaires and the data entry of the returned questionnaires was done by a research institute, independently from the authors, to ensure confidentiality.

### Technology use

Fifteen digital dental technologies were identified as presently available and most relevant to Dutch general dental practitioners, based on the views of experts expressed in interviews [26] and discussion between the authors. During the pilot study no additional technologies emerged. The technologies mentioned in the questionnaire are innovative digital dental technologies and older, more widely used ones. Of these technologies, eight were administration and communication technologies, and seven diagnostic and clinical technologies (see Table 1 for details). Regarding each technology, respondents were asked *Do you use this digital technology*? (yes/no). In addition they were asked if they used any other technology (open question). For every technology they used, three additional questions were posed: *In which year did you start using this technology*? (open question), *Did you or someone else decide to purchase it*? (I, others and I, others than I), and *How satisfied are you with this technology*? which was measured on a 5-point Likert scale (very dissatisfied, mostly dissatisfied, neutral, mostly satisfied and very satisfied). Frequency of use was measured by asking *How often do you use this*? (daily, weekly, monthly, less than monthly or never) for the diagnostic and clinical technologies only. The year of purchase was categorized into three periods after inspection of the data; period 1 (before 2005), period 2 (between 2005 and 2009) and period 3 (between 2010 and the moment of questionnaire completion, spring 2013).

**Table 1:** Description of digital dental technology use among Dutch dentists.

Variables	n (%)	Period ofpurchase*	% purchase per period	Mean satis-faction ± SD	Frequency of use (mode)
**Administrative and communication technologies**					
Digital patient information	233 (93.6)	1	75%	4.3 ± 1.0	
		2	16%		
		3	8%		
Digital agenda	206 (82.7)	1	54%	4.4 ± 0.9	
		2	30%		
		3	16%		
Digital address/financial administration	202 (81.1)	1	74%	4.2 ± 1.0	
		2	17%		
		3	8%		
Practice website	205 (82.3)	1	15%	3.6 ± 1.0	
		2	29%		
		3	56%		
Digital appointments/reminders	86 (34.5)	1	13%	3.8 ± 1.1	
		2	32%		
		3	55%		
Digital information screens	44 (17.7)	1	11%	3.8 ± 0.9	
		2	35%		
		3	54%		
Social media	33 (13.3)	1	-	3.4 ± 0.8	
		2	10%		
		3	90%		
Digital practice supply management	42 (16.9)	1	16%	3.7 ± 0.9	
		2	32%		
		3	51%		
**Clinical and diagnostic technologies**					
Digital intra oral radiography	225 (90.4)	1	44%	4.4 ± 0.9	daily
		2	37%		
		3	19%		
Digital orthopantomogram	143 (57.4)	1	26%	4.4 ± 0.8	weekly
		2	42%		
		3	32%		
Digital 3D radiography (CBCT)	21 (8.4)	1	6%	4.4 ± 0.7	monthly
		2	44%		
		3	50%		
Intra oral camera	65 (26.1)	1	47%	3.7 ± 1.3	daily/weekly
		2	34%		
		3	19%		
Intra oral scanner	30 (12.0)	1	15%	4.0 ± 1.1	daily
		2	27%		
		3	58%		
CAD/CAM system (CEREC)	21 (8.4)	1	20%	4.1 ± 0.9	weekly
		2	45%		
		3	35%		
Digital color determination	17 (6.8)	1	-	3.8 ± 1.2	daily/monthly
		2	53%		
		3	47%		
**Other**	14 (5.6)				

* period 1 = before 2005; period 2 = 2005–2009; period 3 = 2010 to 2013.

Overall technology use was measured in two ways. Respondents were asked to rate their own technology use: *In your opinion*, *do you use more*, *the same amount*, *or less digital technologies than dentists around you*? (more, the same amount, less). Moreover, the sum of technologies each respondent used was calculated, as the total number of the fifteen technologies or ‘other technology’ each respondent used (the number of times a respondent answered ‘yes’). On the basis of results of this sum score, respondents were divided into three groups: low technology users, intermediate technology users and high technology users.

### Personal factors and practice setting

Data regarding age (in years on January 1, 2013), gender, and year of graduation of the respondents were available from previous studies in the panel data (Royal Dutch Dental Association—KNMT). Dentists were asked to indicate whether they worked as a practice owner, independent contractor, employee or ad interim, in either a solo or group practice, an organization or educational institution (closed question with seven options, plus an open-ended ‘other’ option). Subsequently, this was recoded into the variable ‘practice owner’. Respondents who answered they were ‘(shared) owner of a solo or shared practice’, or ‘practice owner in an institution’ were categorized as owners and all others as non-owners. Also, they were asked to indicate in which area(s) of dentistry they worked ((almost) exclusively as a general practitioner, (almost) exclusively as a specialized dentist or as a general practitioner and specialized dentist), as well as which fields of dentistry they had specialized in, if any (nine options: as an endodontist/ gerodontist/ gnathologist/ implantologist/ periodontologist/ pedodontist/ dental anxiety specialist/ specialist in treatment of people with disabilities/ specialist in maxillofacial prosthetics/ other plus an open text field). With the variable ‘specialization’ they were categorized as not having a specialization if they indicated working (almost) exclusively as a general practitioner, and as having a specialization if they answered working as a specialized dentist or as both a general practitioner and a specialized dentist. Furthermore, dentists were asked to indicate chairside (open-ended) and non-chairside working hours (open-ended) with the question *How many hours per week do you work in the practice*, *on average*?Responses were summed to arrive at the variable ‘working hours’, in hours per week. Respondents were also asked how many hours they dedicated on average to three types of professional activity with three open-ended questions (participation in courses and information meetings per year, participation in study groups and inspection per year and reading of dentistry-related literature per month). These values were summed (the last answer multiplied by twelve) yielding the variable ‘professional activities’ in hours per year.

A number of practice characteristics were measured by asking respondents to indicate an estimate of the number of patients attending the practice at least once per year (open question). They were also asked to indicate the number of staff in the practice where they mainly work by filling in the number of persons working in each of the following functions: dentists who are (shared) practice owner, dentist who are not a practice owner, dental or prevention assistants, dental hygienists, secretaries or front office or back office employees, practice managers, system administrators, and other employees (open text field). The first two answers were summed to arrive at the variable ‘number of dentists working in the practice’ and the remaining answers were summed to arrive at the variable ‘number of staff working in the practice’ in number of persons.

### Data analysis

All data were analyzed using IBM Statistical Package for Social Sciences (SPSS) version 21. Descriptive statistics were assessed for each variable. For all statistical tests a significance level of 0.05 was used. Associations between technology use and the other variables were tested using three procedures. Possible differences between technology user groups and categorical variables were assessed using χ² tests. One-way analysis of variance was used when the variables were approximately normally distributed; if not, Kruskall Wallis tests were conducted. In case of p<0.05, these tests were followed by post hoc Tukey’s HSD or Mann-Whitney U tests respectively. Only respondents with valid data on technology use were included in the analysis. Cases with missing data on one of the other variables were excluded from the analysis concerning the relevant variable.

## Results

Of the 1000 dentists in the sample, 246 had responded after the second reminder in June 2013. Of the 1000 questionnaires sent, 45 questionnaires were returned as either undeliverable or the respondent was unable to fill it in for various reasons. 425 dentists of 754 who had not responded to the survey after two reminders were randomly selected and contacted by phone in June 2013 to investigate reasons for non-response. 66 dentists could not be contacted, 249 considered filling in the questionnaire and 110 did not wish to participate. Of the 110 non-respondents, most had no time or no longer wished to participate in surveys (42.7% and 25.5%, respectively). The remaining 31.7% found it too complicated, was not interested in digital technologies, or had other reasons not to participate. 52 of the 110 non-respondents answered follow-up questions, and appeared not to use fewer digital technologies than respondents.

A total of 313 dentists, out of the sample of 1000 dentists, eventually returned the questionnaire, a response rate of 31.3%. 23 of the respondents were no longer working in dental care, and 41 questionnaires were returned incomplete; these were subsequently excluded, leaving 249 questionnaires for further analysis. 65.1% were returned on paper and 34.9% were completed online. Of the respondents 157 (63.1%) were male and 89 (35.7%) female, and of 3 respondents (1.2%) gender and age were unknown. Age ranged between 24 and 64 years. 25 (10.0%) of the dentists were younger than 30, 54 (21.7%) were 30 to 39, 50 (20.1%) were 40 to 49, 82 (32.9%) were 50 to 59 and 35 (14.1%) were 60 to 64 years old. Unpublished data from the Royal Dutch Dental Association (KNMT) for all registered dentists (aged 64 and younger) in the Netherlands in January 2012 shows that the distribution of gender and age group of the sample is highly similar to that of all registered Dutch dentists. This suggests that the sample adequately represents Dutch dental practitioners with regard to these aspects.

### Digital dental technologies in use

The frequencies of use of digital dental technology are presented in Table 1.

Digital registration of patient information is the most frequently used technology (93.2%). Other frequently used administration and communication technologies are a digital agenda (82.4%), practice website (82.0%) and digital address and financial administration (80.8%). With the exception of practice websites, the majority of dentists started using these technologies before 2005. 75% of users started using digital patient information before 2005 (median year 2000), and in the same period 54% of those using a digital agenda started it (median 2004), 74% started using digital address and financial administration systems (median 1998). 15% started using a practice website before 2005, while 56% started it after 2010 (median 2010). Appointments (34.4%), information screens in the waiting area (17.6%), practice supply management (16.8%) and communication about the practice via social media (13.2%) are used digitally with less frequency, and the majority of dentists have started using these during the past three years. 55% of users of digital appointments started to use them after 2010 (median 2010), while in the same period 54% of users of digital information screens started these (median 2010), 90% of those using social media started (median 2012) and 51% started using digital information screens (median 2010).

Of the clinical and diagnostic technologies, digital intra oral radiography (90%) and digital orthopantomograms (57.2%) are used most often, followed by intra oral cameras (26.4%), intra oral scanners (12%), digital 3D radiography (8.4%), digital CAD/CAM (CEREC) systems (8.4%) and digital color determination (6.8). In addition, 5.6% of dentists use other technologies, such as digital pocket registration, hospital equipment, digital cameras, or 3D planning software. Users of digital intra oral radiography started with these mostly up to 2004 (44%; median year 2005); in the same periods use of an intra oral camera was started by 47% (median 2005). From 2005 to 2009, 42% of users started to use digital orthopantomogram systems (median 2007); in the same period 45% started the use of CAD/CAM systems (median 2008) and 53% of the users of digital color determination started (median 2009). 50% of the digital 3D radiography users started (median 2009) as of 2010 up until the moment of the survey, and 58% started the use of intra oral scanners (median 2010) in the same period.

Digital intra oral radiography and intra oral scanners were most often used daily. Digital orthopantomogram systems and CAD/CAM systems were most often used weekly, and digital 3D radiography monthly. Intra oral cameras were most often used daily and weekly, and digital color determination daily and monthly. Mean satisfaction with each digital technology varied between 3.4 and 4.4 (1 = very dissatisfied, 5 = very satisfied), indicating that the respondents were on average satisfied with the technologies they used.

### Total technology use

A total technology use score was calculated, as the sum of digital dental technologies each respondent indicated to use. This varied between 0 and 12, with an average of 6.3 ± 2.3. 1 dentist used no technologies, 4 used two technologies, and all others used multiple digital dental technologies. Most of the dentists who used more than one technology used between four and nine (ranging from 20 to 41 respondents per number of technologies used). Few respondents used more than ten technologies; 8 dentists used eleven digital technologies and 2 used twelve. The score was divided into three groups, based on the number of technologies used, and the frequency with which these were used by all dentists (see the paragraph above). The first group, low technology users (low TU), has adopted between 0 and 4 digital technologies of the most frequently used type, and includes 22.5% of dentists. The intermediate technology users (intermediate TU) have adopted 5 to 7 digital technologies, both very frequently and less frequently used ones (46.2.%); the high technology users (high TU) have 8 to 12 digital technologies, using frequently used technologies and one or more less often used ones (31.3%).

### Technology use by personal characteristics and practice characteristics

In Table 2 personal characteristics are compared by technology user group (TU).

**Table 2:** Digital technology use by personal characteristics and practice characteristics.

Variables		Total n (%)	Low TU n (%)	Intermediate TU n (%)	High TU n (%)	*P*-value^†^
Gender						
	Male	157 (64)	35 (65)	71 (62)	51 (65)	0.895
	Female	89 (36)	19 (35)	43 (38)	27 (35)	
Specialization						
	No	190 (77)	50 (91)	92 (81)	48 (61)	<0.001
	Yes	57 (23)	5* (9)	22 (19)	30* (39)	
Practice ownership						
	Owner	183 (74)	45 (82)	86 (75)	52 (67)	0.131
	Non-owner	64 (26)	10 (18)	28 (25)	26 (33)	
Reply means						
	Paper	162 (65)	40 (71)	78 (68)	44 (56)	0.139
	Online	87 (35)	16 (29)	37 (32)	34 (44)	
Own digital use compared to others						
	More	64 (27)	4* (7)	14* (12)	46* (64)	<0.001
	Same amount	145 (61)	28 (52)	92* (81)	25* (35)	
	Less	30 (12)	22* (41)	7(6)	1* (1)	
Total		249	56	115	78	

^†^χ² Test

*Standardized residuals <-2 or >2.

Dentists’ own evaluation of their digital technology use compared to other dentists was significantly associated with the user groups; the higher the use of digital technologies, the more often dentists indicated that they use it more than others. Whether respondents had replied to the questionnaire by filling in the paper version or the online version was not significantly differently distributed between the TU groups. In all groups, the paper version was the most common means of reply. In the group of high technology users more dentists were specialized than in the group of low users. TU was not significantly associated with gender or with practice ownership.

Mean scores on personal and practice characteristics were tested between the low, intermediate and high technology users (Table 3).

**Table 3:** Distribution of personal and practice characteristics by degree of technology use.

Variable		n	Mean ± SD	*P*-value
Age				
	Low TU	54	50.0 ± 12.6	0.024* c
	Intermediate TU	114	46.4 ± 10.8	
	High TU	78	44.5 ±11.6	
Graduation year				
	Low TU	54	1988 ± 12.7	0.020* c
	Intermediate TU	114	1992 ±10.7	
	High TU	76	1994 ±11.6	
Working hours per week				
	Low TU	51	35.2 ± 8.8	0.003* b, c
	Intermediate TU	100	37.4 ± 9.7	
	High TU	69	41.2 ± 10.3	
Professional activities (hours per year)				
	Low TU	39	193 ±135	0.026^†^ b
	Intermediate TU	92	163 ±137	
	High TU	65	213 ±180	
Patients per year				
	Low TU	48	1750 ± 984	<0.001* a, b, c
	Intermediate TU	94	3132 ± 1710	
	High TU	68	4717 ± 3686	
Persons working in practice				
	Low TU	56	5.9 ± 8.9	<0.001^†^ a, b, c
	Intermediate TU	115	7.6 ± 6.7	
	High TU	76	17.8 ± 16.3	
Dentists working in practice				
	Low TU	42	2.4 ± 6.1	0.001^†^ b, c
	Intermediate TU	98	2.0 ± 1.9	
	High TU	70	4.4 ± 5.3	

*One-way Analysis of Variance with post hoc Tukey HSD test

^†^ Kruskal-Wallis H test with post hoc Mann-Whitney U test

a Low TU- Intermediate TU p<0.05

b Intermediate TU—High TU p<0.05

c Low TU—high TU p<0.05.

Dentists with high TU were significantly younger on average than those with low TU. The same applies to the year in which dentists obtained their degree; dentists with high TU graduated more recently on average than dentists with low TU. High technology users work more hours per week on average than intermediate and low technology users. Dentists with high TU invest averagely more hours per year in professional activities than those with intermediate TU. The mean number of patients attending the practice per year is lowest in the low TU group and highest in the high TU group. The number of people working in a dental practice is lowest when respondents are low technology users and highest when they are high technology users. The mean number of dentists per practice is lower for low TU and intermediate TU than for high TU groups.

## Discussion

This study’s aim was to find out to which extent dentists use digital technologies, looking at dentists’ and dental practices’ characteristics. Our findings suggest that average digital technology use was fairly widespread among dentists, but differs in the degree of use. Overall, administrative and communicative technologies were used more often than diagnostic and clinical technologies, with the exception of intraoral radiography. Three degrees of technology use were distinguished between dentists: low, intermediate and high technology users. These user groups differ on personal factors; high technology users more commonly were of a younger age, graduated more recently, had a specialization, worked more hours per week and spent more time on professional activities. The findings also suggest that dentists working in practices with more patients and with more staff use more digital technologies than those working in smaller practices. Low technology users were averagely older, graduated longer ago, few had a specialization; they had fewer average working hours per week and less patients and staff in the practice than high technology users. Intermediate technology users differed from high technology users in average working hours, time for professional activities, patients per year and staff in the practice.

Technology use and adoption has been widely researched applying social and behavioral science approaches. Many studies describe either actual use [12,23,27] or intended use [10,28] and non-use from the point of view of specific technologies. Yet users [29,30] and non-users [31] differ so much among themselves that they should not be viewed as homogeneous categories. A different angle is to look at groups of adopters or users, identifying the characteristics they share. In ‘diffusion of innovation’ approaches [11] a distinction is made between five adopter groups. Innovators are the first to start adopting an innovation, followed by early adopters. When followed by early majority and late majority groups, adoption becomes fairly widespread. The last group, laggards, long remain non-adopters. These groups may differ in characteristics such as age, innovativeness, and education. In this study we used a similar approach, adapted to emphasize technologies relevant to present-day dental practices. This focus on adoption and use, and associated personal and practice patterns, differs from studies that measure clinical computing in dentistry, which focus more on specific applications and functions of computers [2,17,18]. In a similar way, the use of computers for information seeking has been researched [19,20,32].

High technology users in our study were younger on average than low technology users. The topic of age groups and technology use has been extensively discussed in many papers [33,34]. An influential theory hypothesizes that younger persons, termed ‘digital natives’[33] may be more digitally minded and more inclined to adopt digital technologies than older persons, ‘digital immigrants’. Research on this topic is inconclusive, and some studies suggest that there is no clear generation effect [35–37] and that the terms used for these generational divides are too stark [36]. An alternative explanation that could underlie age differences in technology use is the experience with digital methods of work that younger dentists have gained in their dental education.

Specialized dentists were more often high technology users than non-specialists. A similar association has been found in other health care settings [7,9]. A stronger focus on quality of specific aspects of dental care among specialists, as expected by a number of experts from the dental care field interviewed in an earlier study, may underlie this effect [26]. The higher amount of time used for professional activities among high technology users points in a similar direction.

High technology users in our sample often work in larger practices than low technology users, in line with previous findings across a range of sectors [38,39]. Technologies may yield more tangible results if they can be used more often, as more patients attend and more dentists and other staff use them in a larger practice. Also, investment is likely to be more feasible in larger than in smaller practices. As practices tend to become larger in various countries, such as the Netherlands, it can be expected that digital technologies become increasingly used and increasingly interesting to dentists.

### Strengths and limitations

A self-constructed questionnaire was developed to assess to what extent digital technologies are used by dentists. We found no existing studies that assess the range of common and innovative digital technologies currently used in dental practices, both common and innovative ones. In order to form a measurement of technology use specifically suited for measuring currently present technologies in the dental practice context, construction of the questionnaire was based on interviews with experts in dentistry, dental technology and dental education, and compared with dental and social scientific literature. In order to minimize bias, the questionnaire was furthermore tested in a pilot and discussed by an external research committee.

Studies on computer and internet usage among dentists, conducted in the United States [18], the United Kingdom [17] and in Canada [2], and use of dental technologies in New Zealand [3] found lower adoption levels of some of the digital technologies researched. Digital radiography use especially seems to be much higher among dentists in the present study than among dentists surveyed in earlier studies [2,3,17,18]. In earlier studies, digital radiography was used to a lesser extent than most administration and communication technologies. Remarkably, in the present study the use of intra-oral radiographs especially was comparable to that of digital patient information. In large part this is because digital technology is a rapidly changing field, and thus changed in the years since publication of these papers. Computer usage studies showed that dental administration and communication systems were used more than most clinical and diagnostic technologies [2,17,18], similar to the present study. Not only time, but also location varies. Dental care systems, financial coverage of dental care, as well as institutional settings vary between countries. Perhaps there are cultural differences as well. The role the profitability of dental practices or a government might play in subsidizing investments in sustainable innovations may also have aninfluence on the differences in the implementation of digital tools in different countries. Yet the present study and these earlier papers each suggest that technology use patterns vary between individuals, in association with other factors. Dental education and dental care are facing technological changes in many places, and a wide range of digital dental technologies is finding its way to dentists and dental practices in many advanced economies. Therefore the digital profiles of dentists we found likely accompanies digital technology use in many advanced economies.

This study is based on a sample of dental practitioners in the Netherlands. The response rate (31%) may appear somewhat dissatisfying, but is in line with regular panel surveys among Dutch dentists. To check whether non-response has affected the outcomes, a sample of non-respondents was contacted. As overall technology use of non-respondents appeared not to be lower than that of respondents, and age and gender distribution of the respondents was consistent with that of all registered general practitioners in the Netherlands (unpublished data, KNMT), the findings are considered generalizable to Dutch dentists.

### Concluding remarks

The current state of technology use, as well as the characteristics of dentists and dental practices form the basis for further technological change. For future processes of innovation and implementation of digital technologies to be suitable to dentists and their work, differences in technology use for groups with varying characteristics should be taken into account. Attitudes to digital technologies may further shape these differences, which should be addressed in future research. Developers and suppliers of digital dental technologies and dental educators can benefit from taking these differences into account and adapting communication and training accordingly. For dentists, anticipating the digital trends that are occurring across dental care can lead to better preparation for changes lying ahead, and add to rethinking and weighing the pros and cons of adopting digital technologies to themselves in a wider perspective. Understanding where dentistry is going in terms of digital developments begins with knowing where dentistry stands now, and how digital technologies are incorporated at present.

## References

[1] Lebuis A, Lai B, Emami E, Feine JS. New technologies in health care. Part 1: A moral and ethical predicament. J Can Dent Assoc. 2008;74: 631–635. 18789196

[2] Flores-Mir C, Palmer NG, Northcott HC, Huston C, Major PW. Computer and Internet usage by Canadian dentists. J Can Dent Assoc. 2006;72: 145–145e. 16545175

[3] Tay KI-P, Wu JM-Y, Yet MS-S, Thomson WM. The use of newer technologies by New Zealand dentists. N Z Dent J. 2008;104: 104–108. 18980051

[4] Van Noort R. The future of dental devices is digital. Dent Mater. 2012;28: 3–12. 10.1016/j.dental.2011.10.014 22119539

[5] Gagnon M-P, Desmartis M, Labrecque M, Car J, Pagliari C, Pluye P, et al Systematic review of factors influencing the adoption of information and communication technologies by healthcare professionals. J Med Syst. 2012;36: 241–277. 10.1007/s10916-010-9473-4 20703721 PMC4011799

[6] Venkatesh V, Zhang X, Sykes TA. “Doctors Do Too Little Technology”: A Longitudinal Field Study of an Electronic Healthcare System Implementation. Information Systems Research. 2011;22: 523–546.

[7] Lehoux P, Sicotte C, Denis JL, Berg M, Lacroix A. The theory of use behind telemedicine: how compatible with physicians’ clinical routines? Soc Sci Med. 2002;54: 889–904. 11996023 10.1016/s0277-9536(01)00063-6

[8] Bhattacherjee A, Hikmet N. Physicians’ resistance toward healthcare information technology: a theoretical model and empirical test. Eur J Inf Syst. 2007;16: 725–737.

[9] Ferlie E, Fitzgerald L, Wood M, Hawkins C. The nonspread of innovations: the mediating role of professionals. Acad Manage J. 2005;48: 117–134.

[10] Yarbrough AK, Smith TB. Technology acceptance among physicians: a new take on TAM. Med Care Res Rev. 2007;64: 650–672. 17717378 10.1177/1077558707305942

[11] Rogers EM. Diffusion of Innovations. 5th ed New York: Free Press; 2003.

[12] Fitzgerald L, Ferlie E, Wood M, Hawkins C. Interlocking Interactions, the Diffusion of Innovations in Health Care. Hum Relat. 2002;55: 1429–1449.

[13] Fleuren M, Wiefferink K, Paulussen T. Determinants of innovation within health care organizations: literature review and Delphi study. Int J Qual Heal Care. 2004;16: 107–123. 15051705 10.1093/intqhc/mzh030

[14] Holden RJ. Physicians’ beliefs about using EMR and CPOE: in pursuit of a contextualized understanding of health IT use behavior. Int J Med Inform. 2010;79: 71–80. 10.1016/j.ijmedinf.2009.12.003 20071219 PMC2821328

[15] Nicolini D. The work to make telemedicine work: A social and articulative view. Soc Sci Med. 2006;62: 2754–2767. 16343724 10.1016/j.socscimed.2005.11.001

[16] Flores-Mir C, Palmer NG, Northcott HC, Khurshed F, Major PW. Perceptions and Attitudes of Canadian Dentists toward Digital and Electronic Technologies. J Can Dent Assoc. 2006;72: 243–243e. 16696889

[17] John JH, Thomas D, Richards D. Questionnaire survey on the use of computerisation in dental practices across the Thames Valley Region. Br Dent J. 2003;195: 585–90. 14631436 10.1038/sj.bdj.4810734

[18] Schleyer TKL, Thyvalikakath TP, Spallek H, Torres-Urquidy MH, Hernandez P, Yuhaniak J. Clinical Computing in General Dentistry. J Am Med Inform Assoc. 2006;13: 344–352. 16501177 10.1197/jamia.M1990 PMC1513654

[19] Wårdh I, Axelsson S, Tegelberg A. Which evidence has an impact on dentists’ willingness to change their behavior? J Evid Based Dent Pract. 2009;9: 197–205. 10.1016/j.jebdp.2009.05.002 19913734

[20] Landry CF. Work Roles, Tasks, and the Information Behavior of Dentists. J Am Soc Inf Sci Technol. 2006;57: 1896–1908.

[21] Bjørndal L, Reit C. The adoption of new endodontic technology amongst Danish general dental practitioners. Int Endod J. 2005;38: 52–58. 15606824 10.1111/j.1365-2591.2004.00904.x

[22] Molander A, Reit C, Dahlen G. Reasons for dentists’ acceptance or rejection of microbiological root canal sampling. Int Endod J. 1996;29: 168–172. 9206422 10.1111/j.1365-2591.1996.tb01364.x

[23] Parashos P, Messer HH. The diffusion of innovation in dentistry: A review using rotary nickel-titanium technology as an example. Oral Surg Oral Med Oral Pathol Oral Radiol. 2006;101: 395–401. 16504875 10.1016/j.tripleo.2005.02.064

[24] Locke M, Thomas MB, Dummer PMH. A survey of adoption of endodontic nickel-titanium rotary instrumentation part 1: general dental practitioners in Wales. Br Dent J. 2013;214: E6 1–10. 10.1038/sj.bdj.2013.108 23392051

[25] Rindal DB, Rush WA, Boyle RG. Clinical inertia in dentistry: a review of the phenomenon. J Contemp Dent Pract. 2008;9: 113–121. 18176657

[26] van der Zande MM, Gorter RC, Wismeijer D. Dental practitioners and a digital future: an initial exploration of barriers and incentives to adopting digital technologies. Br Dent J. 2013;215: E21 10.1038/sj.bdj.2013.1146 24309814

[27] Greenhalgh T, Robert G, Macfarlane F, Bate P, Kyriakidou O. Diffusion of Innovations in Service Organizations: Systematic Review and Recommendations. Milbank Q. 2004;82: 581–629. 15595944 10.1111/j.0887-378X.2004.00325.x PMC2690184

[28] Venkatesh V, Morris MG, Davis GB, Davis FD. User Acceptance of Information Technology: Toward a Unified View. MIS Q. 2003;27: 425–478.

[29] Vedel I, Lapointe L, Lussier M-T, Richard C, Goudreau J, Lalonde L, et al Healthcare professionals’ adoption and use of a clinical information system (CIS) in primary care: Insights from the Da Vinci study. Int J Med Inf. 2012;81: 73–87. 10.1016/j.ijmedinf.2011.11.002 22192460

[30] Baron S, Patterson A, Harris K. Beyond technology acceptance: understanding consumer practice. Int J Serv Ind Manag.2006;17: 111–135.

[31] Selwyn N. Digital division or digital decision? A study of non-users and low-users of computers. Poetics. 2006;34: 273–292.

[32] Straub-Morarend C, Marshall T, Holmes D, Finkelstein M. Informational resources utilized in clinical decision making: common practices in dentistry. J Dent Educ. 2011;75: 441–452. 21460265

[33] Prensky M. Digital Natives, Digital Immigrants Part 2: Do They Really Think Differently? Horizon. 2001;9: 1–6.

[34] Morris MG, Venkatesh V. Age differences in technology adoption decisions: implications for a changing work force. Pers Psychol. 2000;53: 375–403.

[35] Bennett S, Maton K, Kervin L. The “digital natives” debate: A critical review of the evidence. Br J Educ Technol. 2008;39: 775–786.

[36] Salajan FD, Schönwetter DJ, Cleghorn BM. Student and faculty inter-generational digital divide: Fact or fiction? Comput Educ. 2010;55: 1393–1403.

[37] Rizzuto TE. Age and technology innovation in the workplace: Does work context matter? Comput Human Behav. 2011;27: 1612–1620.

[38] Cresswell K, Sheikh A. Organizational issues in the implementation and adoption of health information technology innovations: An interpretative review. Int J Med Inform. 2013;82: e73–e86. 10.1016/j.ijmedinf.2012.10.007 23146626

[39] Boonstra A, Broekhuis M. Barriers to the acceptance of electronic medical records by physicians from systematic review to taxonomy and interventions. BMC Heal Serv Res. 2010;10: 231 10.1186/1472-6963-10-231 20691097 PMC2924334

